# A template for multitudes: Germline immune polymorphism of the T cell receptor loci

**DOI:** 10.1016/j.xgen.2022.100231

**Published:** 2022-12-14

**Authors:** Loren Gragert

**Affiliations:** 1 Department of Pathology and Laboratory Medicine, Tulane University School of Medicine, New Orleans, LA, USA

## Abstract

Short-read next-generation sequencing has failed to adequately genotype the T cell receptor (TCR) loci, limiting our ability to characterize the role of germline TCR variation. In this issue of *Cell Genomics*, Rodriguez et al.^1^ describe how a probe-based hybrid capture approach coupled with long-read sequencing can resolve fully phased TCR locus haplotypes from diploid human genomes.

## Main text

In terms of both structural and allelic variation, the genetic regions encoding proteins involved in the adaptive immune response have the greatest complexity in the human genome. The T cell receptor (TCR) loci include more than 200 genes, with multiple gene copies per segment type arrayed in tandem. During T cell development, only one gene per segment is selected to form each TCR. After junctional diversity is introduced between V-D and D-J segments during recombination, millions of unique TCR sequences will comprise each individual’s TCR immune repertoire.

Variability in TCR repertoires between individuals is shaped by both genetic heritability and environment. The TCR’s cognate receptor, the major histocompatibility complex (MHC) protein containing a bound peptide, has a great influence on both genetic and environmental components. The host MHC genotype and an individual’s history of self and foreign peptide antigens displayed within the MHC binding groove will determine which TCR clonotypes are deleted or expanded.^2^

The germline polymorphism of TCR genotypes is another source of immune repertoire variability that has not yet been well characterized. Because of allelic variation and gene copy number variation in the genome, some TCR sequences will be private and potentially unique to a subset of individuals or human populations. Furthermore, the favored patterns of gene usage that generate the naive TCR repertoire may also be influenced by germline polymorphism.^3^

The functional relevance of TCR gene polymorphism in disease is yet not well understood, due to both limitations in DNA genotyping of new samples and deficiencies in the human genome reference. While TCR interactions with their cognate MHC-peptide complexes, are to some extent, degenerate, from the results of early TCR gene association studies it has become clear that not all TCR repertoires have functional equivalence. Targeting genotyping of TCR loci has identified a handful of alleles associated with disease or immune response to infection, but such genetic epidemiology investigations have been far from comprehensive.

The short-read sequencing methods that are most frequently utilized for high-throughput genome sequencing thus far have not thoroughly characterized the human genetic diversity in the TCR region. The TCR region has been especially difficult to genotype accurately using short DNA reads because each read will often map to multiple TCR loci because of high sequence similarity. Structural variation has also been challenging to detect because of inability for short reads to phase between heterozygous positions that are more than a couple hundred bases apart. Long-read sequencing technology promises to overcome both limitations, although there are several technical challenges to producing high-quality sequences.

Rodriguez et al. set about to develop an approach for long-read sequencing of the TCR genomic regions. First, they designed a panel of custom oligonucleotide probes targeting the TRA/D and TRB locus regions of chromosomes 22 and 7 for hybrid capture of long sequence fragments (4–6 kb). After target enrichment, they applied Pacific Bioinformatics HiFi sequencing, which provides high read accuracy. Their approach had previously been successfully applied to the immunoglobulin heavy-chain locus that encodes for antibodies^4^ and was also successful for the TCR loci. Applying this sequencing approach to two family trios and six unrelated donors from diverse populations, they found that the majority of TRA/D and TRB genes could be phased into haplotypes. All types of genomic variation, including SNPs, indels, and structural variants, could be accurately detected.

The long-read sequencing approach had extremely high accuracy in base calling and few limitations in haplotype phasing and coverage. Haplotype phasing was incomplete when there were long runs of homozygosity. Among all samples, only two short regions had gaps in coverage, which might require either higher sequencing depth or alterations in hybridization probe panel design to close the gaps. The researchers compared their results to short-read sequencing on the same samples. Short-read sequencing had a high rate of false positive and false negative SNP calls, likely due to the inability to map short reads to the correct locus. The researchers also identified a false inversion in one of the human genome reference assemblies, indicating applicability in generating more accurate reference and alternative assemblies.

Genetic epidemiology studies in immunogenetics are supported by well-curated reference allele sequence databases and population genetic datasets. Many germline TCR locus variants have not yet been identified by DNA sequencing but instead have been inferred only indirectly by RNA sequencing of recombined TCR transcripts in repertoire analyses.^5^ The study by Rodriguez et al. alone identified 66 novel alleles that could be contributed to the ImMunoGeneTics (IMGT) TR gene sequence database, indicating that major gaps remain in coverage of human genetic diversity. With one or more laboratories established with this long-read sequencing approach, DNA samples could be sent for genotyping to confirm novel alleles and haplotypes and fill in these gaps. A vast increase in alleles and structural variants for TCR loci will soon arrive, similar to the exponential increase in MHC alleles described after next-generation sequencing was applied at scale.^6^

The toolbox for T cell immunology has recently been augmented by increased resources allocated to study the host immune response against SARS-CoV-2. With refinement of high-throughput methods for cloning and characterizing TCRs directed against certain MHC-peptide complexes, we can now observe patterns of TCR gene usage against specific SARS-CoV-2 peptides.^7^ Finally, this work has implications for chimeric antigen receptor T cell immunotherapy, as the researchers identified polymorphisms that could prove to be important for optimizing functionality of CAR-T constructs.

Evolutionary human genetics is another domain that will benefit from improved characterization of TCR diversity. Genetic diversity can be built by having multiple gene copies and by allelic polymorphism within each respective locus. In rhesus macaques, there has been extensive duplication of MHC class II loci leading to a diversity of structural haplotypes in the species.^8^ Similarly, we might expect that TCR gene diversity could have developed differently across species. We don’t yet know whether there has been a balancing selection in human populations that has maintained allele polymorphism in the TCR loci in same way we observe in the MHC.

Improved statistical and informatics approaches are still needed to develop genetic association methods that are fully capable of identifying risk variants in highly variable genomic regions. The TCR and immunoglobulin gene regions may turn out to be major contributors to the missing heritability for immune-mediated disease.^9^ Scientific collaboration between researchers with complementary skillsets will be needed to advance the field. The Adaptive Immune Receptor Repertoire Community of the Antibody Society has been forefront at improving standards for data curation and analysis to support research studies.^10^ The Society for Immune Polymorphism is a nascent effort with similar aims to bring immunogenetics researchers together from across subdisciplines (http://immunepolymorphismsociety.org/). In the same way that genes of the TCR system developed a way to collaborate to form a robust immune response, so, too, will the diverse immunogenetics research community come together to meet new challenges.
